# Hospital-acquired *enterobacteriaceae* Bloodstream Infections in Children

**DOI:** 10.34763/devperiodmed.20192302.131136

**Published:** 2019-07-08

**Authors:** Marta Kłos, Jadwiga Wójkowska-Mach

**Affiliations:** 1 Department of Microbiology, Jagiellonian University, Collegium Medicum, Kraków, Poland

**Keywords:** BSI, incidence, risk factors, drug resistance, zakażenie krwi, zapadalność, czynniki ryzyka, oporność na antybiotyki

## Abstract

*Among the different age groups of children, newborns are most exposed to hospital-acquired bloodstream infection (HA-BS1), especially those who are burdened with additional risk factors, such as low birth weight, immaturity or exposition to medical procedures*.

*The aim of this study was to analyze the aetiology of HA-BS1 among children at high risk, including incidence and drug resistance*.

*The data was obtained from the PubMed database and included medico/ articles as welI as UNICEF and WHO reports published from 2002 to 2017. The study focused on newborns and older children (under 18 years old) with BS1. The main eligibility criteria, aport from age, were Enterobacteriaceae HA-BS1, and the use of invasive medico/ procedures. It was demonstrated that the main risk factors of infection were age and medico/ procedures. Due to non-specific symptoms, sepsis is difficult to diagnose, a fact which leads to a high mortality rate in newborns*.

*The existence of such mufti-drug resistant strains as Extended-Spectrum β-Lactamases (ESBLs) or Carbapenem-Resistant Enterobacteriaceae (CRE) phenotypes is a grave couse for concern*.

## Streszczenie

*Wśród różnych grup wiekowych u dzieci, noworodki należą do grupy najbardziej narażonej na zakażenia krwi (BSI), szczególnie te obciążone dodatkowymi czynnikami ryzyka, takimi jak mola urodzeniowa masa ciała, niedojrzałość lub poddanie procedurom medycznym*.

*Celem opracowania była analiza etiologii zakażeń krwi u dzieci z uwzględnieniem zapadalności i oporności na leki*.

*Dane uzyskane z bazy PubMed obejmowały publikacje medyczne oraz doniesienia UNICEF i WHO ogłoszone w latach 2002-2017. Badaniami objęto noworodki i starsze dzieci (poniżej 18 lat) z BS/. Głównymi kryteriami kwalifikacji, poza wiekiem, było zakażenie krwi poleczkami Enterobacteriaceae oraz stosowanie inwazyjnych procedur medycznych*.

*Przedstawione czynniki ryzyka zakażenia wskazują na szczególne znaczenie wieku oraz procedur medycznych*.

*Zwraca również uwagę wysoka śmiertelność noworodków z powodu posocznicy ze względu na trudności diagnostyczne, spowodowane jej nieswoistymi objawami*.

*Niepokojącyjest fakt występowania także wielu szczepów wielolekoopornych, a wśród nich między innymi fenotypów β-laktamaz o rozszerzonym spektrum działania (ESBL) lub opornych na karbapenemy (CRE)*.

## Introduction

In spite of the development of medicine, newborns’ and children’s health still calls for special efforts and is the cause of concern all over the world. In 2013, ca. 6.3 million live children died before the age of 5 years [1]. According to the WHO, findings concerning perinatal mortality rate during the first 27 days of life [2], the highest incidence is caused by two disease entities, which are responsible for about 25% of deaths. Out of these, sepsis accounts for 14.7% and pneumonia for 10.8%. In Europe these diseases account for 11.3% of overall fatalities, and in Poland for 3.6% – irrespective of birth weight [3]. The risk of neonatal mortality caused by sepsis in combination with very low birth weight (VLBW) is still higher, e.g. in Stoll’s studies conducted by many centers, the mortality rate equaled 37% [4], and in Norwegian studies, it reached 40-43% [5]. Another study confirms age-related differences in morbidity, with the highest index in infants aged 1-11 (156/100000 population), comparing the data with the findings in children aged 5-9 years (22/100000 population) and 10–14 years (20/100000 population) [6]. It should be stressed that sepsis as a cause of death is listed as third on the list, following the preterm birth and perinatal complications [7].

According to the WHO, the term “sepsis” is used for an infection caused by bacteria, viruses, fungi or parasites, when the body response damages tissues and organs [8]. The bacteria that cause BSI have been identified by way of isolating pathogenic microorganisms from at least one aerobic/ anaerobic set of blood culture bottles. Laupland confirms an episode of BSI by isolating one or more pathogens in 48 hours [9], whereas Buetti groups positive cultures into episodes of BSI, if they took place not later than 7 days after the last positive culture in the same patient [10]. Researchers agree that the main pathogens causing sepsis from the Enterobacterales family are *E. coli* and *Klebsiella* sp. [11, 12, 13], whereas some species, such as *Serratia* sp. and *Enterobacter* sp., are so rare (for example in Polish studies, >2.5% and 4.2%, respectively) that there are no grounds for doing a drug resistance study [11, 12, 14].

Bacteremia has been regarded as hospital-acquired if the initial culture was obtained at >48 h on admission to hospital [15]. According to the multicenter programme of monitoring in Europe, in order to confirm BSI in newborns with a VLBW, one should note at least two of the following symptoms:

– body temperature of >38°C or <36.5°C or unstable temperature, tachycardia or bradycardia, apnea, prolonged capillary refill time, metabolic acidosis, hypoglycemia;
– and one of the following parameters of inflammation: C-reactive protein >2.0mg/dl, the I/T ratio of neutrophils (I/T ratio) >0.2, leukocytes <5000/μl and platelets <10 000/μl [16].

Some researchers quote more non-specific symptoms of sepsis in newborns, such as respiratory failure including cyanosis, difficulties in feeding, lethargy or irritability, hypotonia, convulsions, bulging fontanel, poor perfusion, abdominal distension, liver enlargement, and hypothyroidism [17]. Children with hypoxia and acidosis may additionally manifest the symptoms of pneumonia and meconium aspiration syndrome [17]. Unfortunately, BSI as a diagnostic criterion presents with either hyperthermia or hypothermia, but in neonatology, where patients’ body temperature is often controlled in incubators, unstable temperature is easily noticeable [18]. Sepsis in newborns is classified as “early onset” (EOS) if it occurs within the first days of life, and “late onset” (LOS) if it occurs between the fourth day and the end of the infant period.

The aim of our study was to review and summarize some published data that most closely describe the epidemiology of HA*-Enterobacteriaceae* BSI among children at high risk, with an emphasis on incidence and drug resistance.

## Methods

We searched the PubMed, UNICEF and WHO database for English-language studies, and also UNICEF and WHO reports published from 2002 to 2017. The main search criteria in databases, apart from age, were *Enterobacteriaceae* bloodstream infections, and particular exposition to medical procedures. We also used our own research of 2009-2015 on newborns with low birth weight. The review included newborns and older children (under 18 years) with BSI. In our study, after database review, the following were accepted for analysis: 26 hospital-acquired infections (HAI-BSI), 3 community-acquired BSI infections and 5 other types of studies, mainly mixed. In the course of the database search, the changes introduced in 2016 in the taxonomy of the *Enterobacteriaceae* family and the order *Enterobacterales* were not taken into account [19].

## Results and discussion

### Risk factors

The most important risk factors that affect the incidence of sepsis are the following: age, scope of immunizations, and exposition to invasive medical procedures. In addition to the aforementioned causes, Folgori adds others, such as: immunodeficiency, kidney failure, transplantations, carcinomas, and preterm birth [14].

A newborn’s passive immunological response is based on an array of maternal antibodies of the IgG isotype transferred still in the prenatal development, therefore one frequently finds a higher level of antibodies in a full-term newborn than in a woman after delivery, but they usually disappear after several months, while in preterm newborns this process is much faster [20]. An active immunological response in newborns with low birth weight is insufficient both with respect to bacterium opsonization ability, neutrophil activity, and the activity of other components of the serum, such as antibodies, cytokines, and acute-phase proteins [21].

Other important elements which considerably burden newborns are hospitalization and intensive multiple medical invasive procedures associated with neonatal care (particularly in the case of newborns with low birth weight), such as the administration of corticosteroids in infants with impaired breathing that lowers their immunological response [22] and the necessity to use diagnostic and therapeutic invasive procedures (endovascular catheters, assisted ventilation, and parenteral nutrition). All of these are important risk factors in the development of infections. A long-term stay at hospital and neonatal intensive care also increase the risk of the development of undesirable microorganisms; this is enhanced by overcrowded wards, the insufficient number of personnel and their being overworked. In the United States, the average stay of newborns with birth weight <800 grams is 112 days, with the central catheter (including the umbilical catheter) and mechanic ventilation being used in the group of preterm newborns on average throughout 50% of the days the preemies stay at NICUs (Neonatal Intensive Care Units) [23, 24, 25]. In Poland, the average stay of the newborn who has not been diagnosed with infections associated with low birth weight <800 grams is much longer: 164 days [26]. Researchers’ studies confirm that the administration of steroids or other immunosuppressive medicines for 2 weeks and longer as well as a prolonged stay at a hospital are factors predisposing to infection, especially HA-BSI [26, 27]. The interesting fact is that the presence of the endovascular or urinary catheter was not an essential or immediate cause of the development of infection, although in the majority of cases most patients had catheters at the moment of infection [27]. Moreover, two independent findings indicate that a high risk of infection associated with health care is present among infants younger than 12 months, those who are terminally ill, with a long-term stay at hospital wards and those given treatment using invasive medical devices [28, 29].

Other findings indicate risk factors associated with HA-BSI as similar to those described in the population of Polish newborns – low gestational age, low birth weight, surgery [30, 31], the presence of an invasive device, longterm stay, central venous catheters or mechanic ventilation [29]. Laupland stresses that the BSI risk was highest in newborns and was considerably reduced after the first year of life [9].

According to Li’s and Dong’s studies, children with additional burdens are vulnerable to *E. coli* bloodstream infection. They show the following risks: preterm birth, neonatal respiratory distress syndrome and newborn asphyxia [32, 33]. In Poland it was proved that natural delivery is an additional burden [31].

Specific factors, such as breastfeeding, increases the risk of cross-transmission of microorganisms. In older children toys are also associated with a higher risk of infection connected with health care. If there is contact between children in hospitals and their parents and brothers or sisters, with uncontrolled secretions, or the flow of body fluids, there are extensive risks for the spreading of infection. Pediatric patients and adults share common risk factors for infection associated with hospitalization, including exposition to endovascular catheters, hyper alimentation, mechanic ventilation, and coexistent diseases, e.g. ones weakening the immune system. Exposition to the BSI can be reduced, evidence of which is provided by findings of the NACHRI (National Association of Children’s Hospitals and Related Institutions), where the reduction of CVC-BSI (central venous catheter-related bloodstream infection) by 41% was achieved [18].

## *HA-enterobacteriaceae* BSI - incidence and drug resistance

### BSI in newborns

In the late 1990s, it was observed by Hamer et al that the most frequent species from the Enterobacteriaceae family in the case of BSI were *Klebsiella* spp. and *Escherichia coli* [12]. This observation is the result of multicenter studies on neonatal infections with very low birth weight infants as part of the American NHSN program, which in 1986-1994 studied early infection by maternally acquired Group B *Streptococcus* (GBS) dominated *Streptococcus agalactiae*, followed by E. *coli* [12, 34].

The prevention of early GBS infections and induction of screening tests caused a change in the microbiology of neonatal infections. Currently, according to Stoll, *E. coli* is the most frequent pathogen in preterm infants, while GBS remains the most frequent pathogen in children born on time [35]. Unfortunately, among preterm infants, prophylaxis of *E.coli* sepsis is very difficult [35]. Not all maternally acquired risk factors can be limited by prevention procedures; there are acute or chronic inflammations, especially inflammation of the amnion, which dramatically increase the incidence and case fatality of infections, including ones by *E. coli* [31].

Also, the most recent review of neonatal contracted sepsis gives similar results – in newborns and infants <60 days of age, the most frequent microorganisms are *E. coli* (12.2%) and *Klebsiella* spp. (11.6%) [12]. A considerable part of the *E. coli* isolates was resistant to cotrimoxazole and antibiotics from the β-lactams group, like ampicillin or later generation cephalosporins; moreover, an additional cause for concern was the presence of moderately resistant *Enterobacteriaceae* bacteria highly resistant to various antimicrobial means, including gentamycin, and ciprofloxacin [12, 13]. The Swiss studies of 2008-2014, comprising 20 hospitals, prove that non-E *coli Enterobacteriaceae* (8.7 %) dominate in newborns [10]. The BSI episodes caused by *Klebsiella* spp. numbered 3.3 % of all BSI episodes, whereas the resistance indicators for *E. coli* in the study were below 10 % for aminoglycosides, β-lactam antibiotics (third-/fourth generation cephalosporins) and β-lactam antibiotics associated with β-lactamase inhibitor (e.g. piperacillin-tazobactam) [10]. β-lactam antibiotics associated with β-lactamase inhibitor are more effective against resistant strains, as indicated by Betti's studies where resistance to amoxicillin–clavulanic acid was observed in 27 % cases [10]. A current comparison with *E. coli*, found that the indicators of resistance in non-E *coli Enterobacteriaceae* were higher for all antibiotics [10]. In Poland, a major problem for neonates are the strains with ESBL phenotype, which make up 29% *Enterobacteriaceae* in Polish NICUs [11]. The highest percentage of resistance among Polish neonates was observed against β-lactams (amoxicillin 91.7-100%; amoxicillin-clavulanic acid 54.2-56.3%), aztreonam (81.3%), and cephalosporins (56.3%) [11].

As far as community-acquired infections in the neonatal period (0-28 Days) are concerned, Gram-negatives bacteria predominated in aggregated data (Gramnegative to Gram-positive ratio 1.6:1). The literature that has been published since 1990 from the studies in those developing countries which reported resistance to serious community-acquired infections (including sepsis, pneumonia, and meningitis) shows that among neonatal pathogens a high ratio of *E. coli* was resistant to ampicillin (72%) and cotrimoxazole (78%) [36]. In very early onset sepsis, there is a domination of *Klebsiella* sp., causing 26% of all infections, whereas in early onset sepsis – *Klebsiella* sp. accounted for 25%. Generally, the ratio between Gram-negative and Gram-positive was 2:1 [37]. It follows from the studies of 1996-2007 that almost all *Klebsiella* spp. isolates were resistant to β-lactams – ampicillin (97%), and the third generation cephalosporins (66%); there was also a high percentage of resistance to cotrimoxazole (45%) [36]. Shaver's studies confirm the uniform resistance of *Klebsiella* sp. to ampicillin [36]. Furthermore, many authors consider *Klebsiella* sp. congenitally resistant to ampicillin, but on the other hand, EUCAST (The European Committee on Antimicrobial Susceptibility Testing) recommends that the antibiogram prepared for the Enterobacterales family (including *Klebsiella* sp.) should include ampicillin and ampicillin with sulbactam [http://www.eucast.org/eucast_news/news_singleview/?debug=1&tx_ttnews%5Btt_news%5D=2998rcHash=c3fa88f825754f85fe33dl606abaf083] Resistance to gentamicin was low among *E. coli* (13%), but considerably higher to *Klebsiella* sp. (60%); moreover, increased resistance to third generation cephalosporins with *Klebsiella* sp. and *E. coli* was noted [36]. Resistance of *Klebsiella* sp. and *E. coli* is usually attained via plasmid-mediated ESBL production. Owing to the presence of other resistance-conferring genes on these movable plasmids, such organisms are also resistant to other drugs, including aminoglycoside antibiotics.

### BSI in older hospitalized children

In pediatric intensive care units (PICUs), the incidence of HA-BSI was inversely proportional to age [15]. Researchers report a higher mortality rate in children infected with microorganisms that are G-negative (36%) or fungi (32%) [18]. Other studies confirm that Enterobacteriaceae BSI occurred more often in newborns [32]. In all children, the isolation of *Enterobacter* sp. and *Klebsiella* sp. considerably increased over years, nevertheless the youngest group had the highest incidence rate [28]. In the years of 2011-2012 HA-BSI were the most frequent types of infections – alongside lower respiratory tract infection (45% infections) [29]. The incidence of BSI was most common in PICUs, while Enterobacteriaceae were most often isolated in NICUs [29]. As regards the group of microorganisms that is most frequently isolated, Zing's findings can be compared with the findings in French, Swiss, and American studies [29]. In the observations of 15 French hospitals, it has been proved that the most frequent source of secondary BSI were urinary tract infections (66.2%) and gastrointestinal infections (19.5%); the mortality rate equaled 17%; 16.7% children were transferred to ICU and 9.5% died during observation [13]. In French hospitals the isolates of *E. coli* in HA-BSI were generally resistant to β-lactams: amoxicillin (63.1%) and the third generation cephalosporins (3.6%); vulnerable to ofloxacin and gentamicin (96.4% and 96.4%); 34.5% of strains manifested resistance to cotrimoxazole. 3.6% were multidrug-resistant [13].

Exposition to ESBL (extended-spectrum β-lactamase-producing) *Escherichia coli* and *Klebsiella* spp. infection particularly increases due to prior antimicrobial therapy – extended-spectrum penicillins, aminoglycosides, and cotrimoxazole [27]. In the American studies of 2010-2011 comprising 55 institutions and analyzing pediatric antibiograms, it was also noted that the activity of ampicillin associated with β-lactamase inhibitor sulbactam against G-negative intestine pathogens was worse than the majority of other β-lactamase, whereas 6 places reported specific ESBL for *E. coli* and *K. pneumoniae* [38]. Studies in the pediatric ICUs in Italy and Brazil showed 45% ESBL isolates among all *Enterobacteriaceae* isolates [14]. Besides the presence of β-lactamase, it has also been confirmed that there is resistance to carbapenems (Carbapenem-Resistant *Enterobacteriaceae*, CRE): 2% [14], and fortunately CRE infections are still relatively rare in hospitalized children, their incidence being reported as lower than 1% [14]. Carbapenems manifested higher activity against the majority of G-negative bacteria also in the United States [38].

## Conclusions

HA - *Enterobacteriaceae* BSI leads to the highest rate of incidence of HA-infections and mortality rates among newborns. Probably one of the most important risk factors for newborns are perinatal complications requiring hospitalization or community infections, with severe courses also requiring hospitalization.

Due to many non-specific symptoms of sepsis, it is difficult to diagnose, especially in preterm babies or newborns with very low birth weight, a fact that may delay the process of treatment and increase the rate of HA-BSI mortality.

The major and most frequently reported risk factors of HA-BSI are age (preterm birth), and exposition to invasive medical procedures.

The most common aetiology of HA-BSI are *E. coli* and *Klebsiella* sp. The Enterobacterales show a high resistance to β-lactams, also in association with an inhibitor, and a high percentage of isolates are ESBL and CRE phenotypes. In the treatment of very young patients, it is necessary to act very carefully in the selection of antibiotics for empirical therapy.

## References

[1] You D, Bastian P, Wu J Levels and trends in child mortality.

[2] Black RE, Cousens S, Johnson HL (2010). Global, regional, and national causes of child mortality in 2008: a systematic analysis. Lancet.

[3] (2010). Central Statistical Office: Demographic year book of Poland.

[4] Stoll BJ, Hansen NI, Bell EF (2002). Changes in pathogens causing early-onset sepsis in very-low-birth-weight infants. N Engl J Med.

[5] Ronnestad A, Abrahamsen TG, Medbo S (2005). Late-onset septicemia in a Norwegian national cohort of extremely premature infants receiving very early full human milk feeding. Pediatr.

[6] Watson RS, Carcillo JA, Linde-Zwirble WT (2003). The epidemiology of severe sepsis in children in the United States. Am J Respir Crit Care Med.

[7] Liu L, Oza S, Hogan D (2015). Global, regional, and national causes of child mortality in 2000–13, with projections to inform post-2015 priorities: an updated systematic analysis. Lancet.

[8] Skvortsova V Seventieth World Health Assembly. International Health Regulations.

[9] Laupland KB, Gregson DB, Vanderkooi OG (2009). The Changing Burden of Pediatric Bloodstream Infections in Calgary, Canada, 2000=2006. Pediatr Infect Dis J.

[10] Buetti N, Atkinson A, Kottanattu L (2017). Patterns and trends of paediatric bloodstream infections: a 7-year surveillance study. Eur J Clin Microbiol Infect Dis.

[11] Chmielarczyk A, Pobiega M, Wojkowska-Mach J (2015). Bloodstream Infections due to Enterobacteriaceae Among Neonates in Poland – Molecular Analysis of the Isolates. Pol J Microbiol.

[12] Hamer DH, Darmstadt GL, Carlin JB (2015). Etiology of Bacteremia in Young Infants in Six Countries. Pediatr Infect Dis J.

[13] Burdet C, Clermont O, Bonacorsi S (2014). Escherichia coli Bacteremia in Children. Age and Portal of Entry Are the Main Predictors of Severity. Pediatr Infect Dis J.

[14] Folgori L, Bernaschi P, Piga S (2016). Healthcare-Associated Infections in Pediatric and Neonatal Intensive Care Units: Impact of Underlying Risk Factors and Antimicrobial Resistance on 30-Day Case-Fatality in Italy and Brazil. Infect Control Hosp Epidemiol.

[15] Gray J, Gossain S, Morris K (2001). Three-year survey of bacteremia and fungemia in a pediatric intensive care unit. Pediatr Infect Dis J.

[16] Commission Decision of 28 April 2008 amending Decision 2002/253/EC laying down case definitions for reporting communicable diseases to the Community network under Decision No 21 19/98/EC of the European Parliament and of the Council (notified under document number C (2008) 1589) (Text with EEA relevance) 2008/426/EC.

[17] Shah BA, Padbury JF (2014). Neonatal sepsis. An old problem with new insights. Virulence.

[18] Posfay-Barbe KM, Zerr DM, Pittet D (2008). Infection control in paediatrics. Lancet Infect Dis.

[19] Adeolu M, Alnajar S, Naushad S, Gupta RS (2016). Genome based phylogeny and taxonomy of the “Enterobacteriales”: proposal for Enterobacterales ord. nov. divided into the families Enterobacteriaceae, Erwiniaceae fam. nov., Pectobacteriaceae fam. nov., Yersiniaceae fam. nov., Hafniaceae fam. nov., Morganellaceae fam. nov., and Budviciaceae fam. nov. Int J Syst Evol Microbiol.

[20] Van Der Zwet WC, Vandenbroucke- Grauls CM, van Elburg RM (2002). Neonatal antibody titers against varicella-zoster virus in relation to gestational age, birth weight, and materna titer. Pediatr.

[21] Wynn JL, Neu J, Moldawer LL (2009). Potential of immunomodulatory agents for prevention and treatment of neonatal sepsis. J Perinatol.

[22] Bancalari E (1998). Corticosteroids and neonatal chronic lung disease. Eur J Pediatr.

[23] Gladstone IM, Ehrenkranz RA, Edberg SC (1990). A ten-year review of neonatal sepsis and comparison with the previous fifty-year experience. Pediatr Infect Dis J.

[24] (2004). National Nosocomial Infections Surveillance (NNIS): System Report, data summary from January 1992 through June 2004, issued October 2004. Am J Infect Control.

[25] Geffers C, Baerwolff, Schwab F (2008). Incidence of healthcare-associated infections in high-risk neonates: results from the Germen surveillance system for very-low-birth weight infants. J Hosp Infect.

[26] Rozanska A, Wojkowska-Mach J, Adamski P (2015). Infections and risk-adjusted length of stay and hospital mortality in Polish Neonatology Intensive Care Units. Int J Infect Dis.

[27] Zaoutis TE, Goyal M, Chu JH (2005). Risk Factors for and Outcomes of Bloodstream Infection Caused by Extended-Spectrum β-Lactamase–Producing Escherichia coli and Klebsiella Species in Children. Pediatry.

[28] Henderson KL, Johnson AP, Muller-Pebody B (2010). The changing aetiology of paediatric bacteremia in England and Wales, 1998-2007. J Med Microbiol.

[29] Zingg W, Hopkins S, Gavet-Ageron A (2017). Health-care-associated infections in neonates, children, and adolescents: an analysis of paediatric data from the European Centre for Disease Prevention and Control point-prevalence survey. Lancet. Infectious Diseases.

[30] Wojkowska-Mach J, Borszewska-Kornacka M, Domanska J (2014). Late-Onset Bloodstream Infections of Very-Low-Birth-Weight Infant. Data from the Polish Neonatology Surveillance Network in 2009-2011. BMC Infect Dis.

[31] Chmielarczyk A, Wojkowska-Mach J, Romaniszyn D (2014). Mode of delivery and other risk factors for Escherichia coli infections in very low birth weight infants. BMC Pediatr.

[32] Dong L, Zhang XY, Li CC (2017). Characteristics of epidemiology and antimicrobial resistance of gram-negative bacterial bloodstream infections in children. Zhonghua Er Ke Za Zhi.

[33] Li S, Guo L, Liu L (2016). Clinical features and antibiotic resistance of Escherichia coli bloodstream infections in children. Zhonghua Er Ke A Zhi.

[34] Gaynes RP, Edwards JR, Jarvis WR (1996). Nosocomial infections among neonates in high-risk nurseries in the United States. Pediatrics.

[35] Stoll BJ, Hansen NI, Sanchez PJ (2011). Early onset neonatal sepsis: the burden of group B streptococcal and E. coli disease continues. Pediatrics.

[36] Thaver D, Ali SA, Zaidi AKM (2009). Antimicrobial Resistance Among Neonatal Pathogens in Developing Countries. Podiatry Infect Dis J.

[37] Zaidi AKM, Thaver D, Assad Ali S (2009). Pathogens Associated with Sepsis in Newborns and Young Infants in Developing Countries. Pediatr Infect Dis J.

[38] Tammi PD, Robinson GL, Gerber JS (2013). Paediatric Antimicrobial Susceptibility Trends across the United States. Infect Control Hosp Epidemiology.

